# Tenofovir disoproxil fumarate in pregnancy for prevention of mother to child transmission of hepatitis B in a rural setting on the Thailand-Myanmar border: a cost-effectiveness analysis

**DOI:** 10.1186/s12884-021-03612-z

**Published:** 2021-02-22

**Authors:** Marieke Bierhoff, Chaisiri Angkurawaranon, Marcus J. Rijken, Kanlaya Sriprawa, Pachinee Kobphan, Francois N. Nosten, Michèle van Vugt, Rose McGready, Angela Devine

**Affiliations:** 1 Shoklo Malaria Research Unit, Mahidol-Oxford Tropical Medicine Research Unit, Mahidol University, Mae Sot, 63110 Thailand; 2 Division of Infectious Diseases, Academic Medical Center, University of Amsterdam, Amsterdam, The Netherlands; 3 Department of Family Medicine, Faculty of Medicine, Chiang Mai University, Chiang Mai, 50200 Thailand; 4 Department of Obstetrics and Gynaecology, Amsterdam UMC, University of Amsterdam, Amsterdam, the Netherlands; 5 Centre for Tropical Medicine and Global Health, Nuffield Department of Medicine Research Building, University of Oxford, Oxford, OX3 7FZ UK; 6 Division of Global and Tropical Health, Menzies School of Health Research, Charles Darwin University, Casuarina, Australia; 7 Centre for Epidemiology and Biostatistics, Melbourne School of Population and Global Health, The University of Melbourne, Parkvilles, Australia

**Keywords:** Cost-effectiveness, Perinatal infection, Antiviral therapy

## Abstract

**Background:**

Hepatitis B Virus (HBV) is transmitted from mother to child which can be prevented via birth dose vaccine combined with three follow up hepatitis B vaccines, hepatitis B immunoglobulins (HBIG), and maternal antiviral treatment with Tenofovir Disoproxil Fumarate (TDF). This study evaluates the cost effectiveness of six strategies to prevent perinatal HBV transmission in a resource limited setting (RLS) on the Thailand-Myanmar border.

**Methods:**

The cost effectiveness of six strategies was tested by a decision tree model in R. All strategies included birth and follow up vaccinations and compared cost per infection averted against two willingness to pay thresholds: one-half and one gross domestic product (GDP) per capita. Strategies were: 1) Vaccine only, 2) HBIG after rapid diagnostic test (RDT): infants born to HBsAg+ are given HBIG, 3) TDF after RDT: HBsAg+ women are given TDF, 4) TDF after HBeAg test: HBeAg+ women are given TDF, 5) TDF after high HBV DNA: women with HBV DNA > 200,000 are given TDF, 6) HBIG & TDF after high HBV DNA: women with HBV DNA > 200,000 are given TDF and their infants are given HBIG. One-way and probabilistic sensitivity analyses were conducted on the cost-effective strategies.

**Results:**

*Vaccine only* was the least costly option with *TDF after HBeAg test* strategy as the only cost-effective alternative. *TDF after HBeAg test* had an incremental cost-effectiveness ratio of US$1062; which would not be considered cost-effective with the lower threshold of one-half GDP per capita. The one-way sensitivity analysis demonstrated that the results were reasonably robust to changes in single parameter values. The PSA showed that *TDF after HBeAg test* had an 84% likelihood of being cost effective at a willingness to pay threshold of one GDP per capita per infection averted.

**Conclusions:**

We found that *TDF after HBeAg test* has the potential to be cost-effective if TDF proves effective locally to prevent perinatal HBV transmission. The cost of TDF treatment and reliability of the RDT could be barriers to implementing this strategy. While *TDF after RDT* may be a more feasible strategy to implement in RLS, *TDF after HBeAg test* is a less costly option.

**Supplementary Information:**

The online version contains supplementary material available at 10.1186/s12884-021-03612-z.

## Background

Hepatitis B virus (HBV) is highly endemic in South East Asia (SEA) and the predominant mode of infection is mother to child transmission (MTCT) [1, 2]. MTCT can mostly be prevented by the administration of passive immunoprophylaxis with hepatitis B Immunoglobulin (HBIG), in combination with active immunoprophylaxis by vaccination at birth (birth dose, BD), and approximately at 2, 4 and 6 months of age [3]. Nonetheless transmission still occurs with optimal preventive strategies, in an estimated 8–32% of cases of hepatitis B envelope antigen (HBeAg) positive cases [1, 4]. Prevention of HBV transmission in a resource limited setting (RLS) is challenging as HBIG is not widely available and childhood vaccination coverage is suboptimal [5, 6]. Moreover, birth in the RLS have traditionally been at home, which precludes timely HBV BD vaccination. Infants born in the night may present to the clinic soon after, in the next few days.

Treatment with antiviral therapy during pregnancy is one strategy under consideration to reduce HBV MTCT. Antivirals that are active against HBV like tenofovir disoproxil fumarate (TDF) may reduce the risk of MTCT by reducing the HBV DNA during pregnancy to preferably undetectable at the time of delivery and this might be a more feasible option than HBIG in a RLS [7, 8]. Provision of TDF in the third trimester of pregnancy reduces the HBV DNA significantly [4, 9–11], but still require HBIG to ensure prevention. However, earlier administration of TDF during pregnancy, at 24–27 weeks gestation or earlier, may achieve better suppression compared to starting in third trimester [11, 12]. Although antivirals may be available in RLS to treat patients with human immunodeficiency virus (HIV) or HIV/HBV coinfection, few (if any) programs cover the expense of these medications in patients with HBV monoinfection [13]. While it could be obtained from out of pocket expenses this would be beyond the means of this population. In studies of HBV mono-infected populations, antivirals in combination with immunoprophylaxis provides better prevention of MTCT compared to active or passive immunoprophylaxis alone [14–16]. In these studies, the pregnant women were screened for the presence of HBeAg presence and/or HBV DNA and only these women were prescribed antiviral treatment in their third trimester of pregnancy. This strategy of combined active and passive immunoprophylaxis and maternal TDF from third trimester has been proven to be cost effective in high income countries; but in most RLS, HBV DNA testing is not available and in some cases HBeAg testing is not feasible [16, 17].

Published models of TDF in pregnancy are all from African or western settings where antivirals are used in HIV treatment (supported via government programs) and have a lower HBV prevalence compared to SEA [13, 14, 18]. Furthermore, the models do not account for potential side effects of short term treatment with antivirals, like hepatitis flare after cessation following longer duration of treatment. Checking for hepatic flares means the women would need additional blood tests and have a possibility of prolonged antiviral treatment.

The purpose of this study was to evaluate the potential cost effectiveness of HBV prevention in clinics on the border of Thailand with Myanmar where pregnant women have a high prevalence of monoinfection with HBV. This rural, RLS currently does not have routine access to HBeAg, HBV DNA testing or HBIG.

## Methods

### Setting

Shoklo Malaria Research Unit (SMRU) provides humanitarian health care for marginalized populations on the border of Thailand and Myanmar. At the time of data collection, antenatal care and delivery services were available at two migrant sites, Mawker Thai (MKT) and Wang-Pha (WPA), and one refugee site, Maela (MLA) camp. Attendance at these clinics is free. Public health programmes, including childhood vaccinations, supported in part by the Thailand Department of Public Health. Thai guidelines recommend HBIG for all HBsAg positive patients, but unless payment can be guaranteed this is not provided to non-Thai mothers. SMRU has not had funding available for HBIG since 2016.

### Model structure and strategies for prevention of HBV transmission

**Fig. 1:**
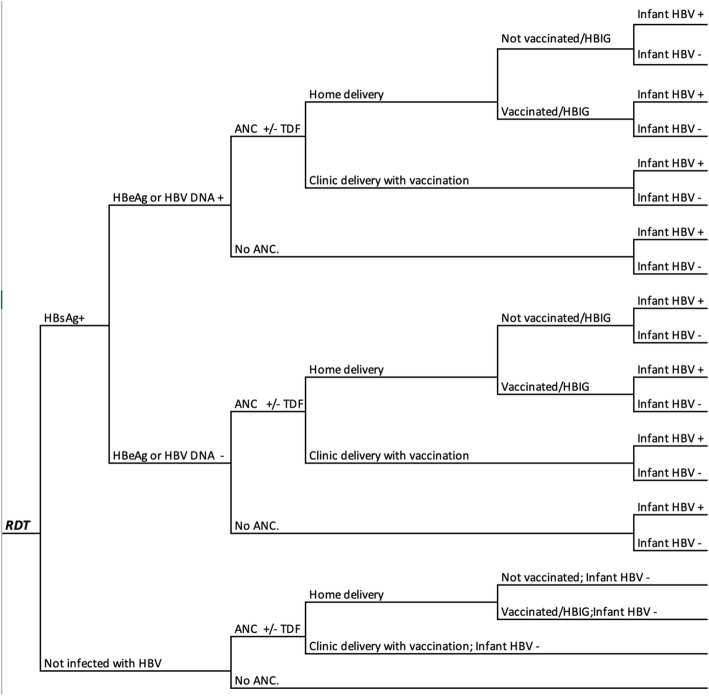
Schematic design of the decision tree models. ANC, antenatal care; DNA, Deoxyribonucleic acid; HBeAg, hepatitis B envelope antigen; HBIG, hepatitis B immunoglobulins; HBV hepatitis B virus; RDT, HBsAg; HBsAg, hepatitis B surface antigen; Rapid Diagnostic test; TDF, Tenofovir Disoproxil Fumarate

A decision tree model for the cost-effectiveness per perinatal infection averted of HBV infection was adapted from a previously described study in this setting [19] with R statistical software [20] using a health care provider perspective (Fig. 1). The model structure for each strategy is shown in additional files 1, 2, 3, 4, 5 and 6. The decision tree uses a time horizon that begins with first prenatal contact through to six months post partum (when the last of the three follow-up vaccinations is provided). The long-term costs and effects of HBV on the mother and child were not included.

Six strategies utilizing three different diagnostic tests were included in the economic evaluation. The diagnostic tests included a point-of-care rapid diagnostic test (RDT) (One Step Bioline hepatitis B Surface Antigen Test Strip, Pacific Biotech) and two off-site tests: a confirmatory test for HBeAg status (HBeAg electrochemiluminescence immunoassay, Roche Diagnostics, USA) and a molecular HBV PCR test to determine HBV DNA (HBV DNA assay, Fast Track Diagnostics). Six strategies were evaluated for a cohort of 3200 pregnant women (the average annual number seen at SMRU in 2012–2016), regardless of age (Table 1, Additional files 1, 2, 3, 4, 5 and 6 show the model diagrams):

1. *Vaccine only:* HBV vaccination (passive immunization with HepB-BD and three follow up vaccinations) provided to all infants. No maternal HBV screening is involved.
2. *HBIG after RDT:* Maternal HBV screening at the first antenatal visit using the RDT. Infants of mothers who test HBsAg+ during antenatal care or at delivery are given HBIG and vaccinations.
3. *TDF after RDT:* Maternal HBV screening at the first antenatal visit using the RDT. Mothers who test HBsAg+ during antenatal care are given TDF immediately regardless of their estimated gestational age and vaccinations.
4. *TDF after HBeAg test:* Similar to *TDF after RDT* with the additional step of confirmatory testing of HBsAg and HBeAg in those testing RDT positive. Only HBeAg+ women are given TDF.
5. *TDF after high HBV DNA:* Similar to *TDF after RDT* with the additional step of HBV DNA testing in those testing RDT positive (no HBeAg testing). Only women with HBV DNA > 200,000 IU/mL are given TDF.
6. *HBIG & TDF after high HBV DNA:* Similar to *TDF after high HBV DNA* with the addition of HBIG for the infants of those testing with an HBV DNA > 200,000 IU/mL during antenatal care.

**Table 1:** Details of the interventions included in each strategy for the prevention of perinatal hepatitis B transmission

	*Vaccine only*	*HBIG after RDT*	*TDF after RDT*	*TDF after HBeAg test*	*TDF after high HBV DNA*	*HBIG & TDF after high HBV DNA*
Vaccination of all infants born in the clinic or presenting within 12 h after birth	X	X	X	X	X	X
Screening for HBsAg with RDT		X	X	X	X	X
Confirmatory HBeAg test during ANC				X		
HBV DNA test during ANC					X	X
HBIG for infant		X				X
Maternal TDF until 1 month post partum			X	X	X	X

*ANC* antenatal care, *DNA* Deoxyribonucleic acid, *HBV* hepatitis B Virus, *HBIG* hepatitis B immunoglobulin, *HBeAg* hepatitis B envelope antigen, *HBsAg* hepatitis B surface antigen, *RDT* rapid diagnostic test, *TDF* Tenofovir Disoproxil Fumarate, Vaccination, includes birth dose and 3 follow up vaccinations

Since TDF following positive HIV testing is routinely included in the antiviral regimen in pregnant women, this model only included women who tested HIV-negative. Several further assumptions were made in the model. The first assumption was that all infants who received the vaccine at birth also received the second, third and fourth doses of the vaccines. It was assumed that all vaccinations and HBIG (if given) were administered at appropriate times. All pregnancies were assumed to be singleton and result in a liveborn infant that lived until at least six months of age in order to receive all four doses of the vaccine and the HBsAg test of the infant at six months of age. It was assumed that women are fully adherent to their TDF regimen and attend their follow up appointments. Lastly, infants born to mothers that are HBV negative at baseline ANC screen were assumed to be HBsAg negative at birth.

### Probabilities from primary attendance data

Primary attendance data were analysed using SPSS version 23 to calculate means and confidence intervals. For the HBV prevalence data (Table 2) and the attendance data, we used a prospective cohort from the period Aug-2012 to Dec-2016 (*n* = 11,025). In this cohort, 6.2% (687/11,025) were HBsAg+, and 30.7% (211/687) of those were HBeAg+ [21]. Since HBV DNA is not routinely available at this location, it was assumed that 82% of the HBeAg+ women had an HBV DNA of > 200,000 IU/mL [22]. Using HBeAg prevalence data in our cohort, this equates to 25.2% of the HBsAg positive women in our cohort having a high HBV DNA. Vaccination data were derived from children born at the SMRU clinics in 2015 since it was the only year where documentation of vaccination at the hospital of birth was published [5]. In this cohort, birth dose vaccination was documented in 93.0% (1441/1549) and HBIG administration in 76.5% (26/34) of children born to HBeAg + women. The home delivery rate in this population was 13.0% (199/1535). The confirmatory tests were done in an external laboratory of a local tertiary referral hospital in Thailand. In order to have all HBV test results before delivery, women would need to present to the clinics at least seven days before delivery, which happened for 94.6% (95% CI 94.4–99.8%) of the women in our cohort. Table 2 shows all parameters used in the model.

**Table 2:** Parameters including base values, range, distributions, and sources. All costs are in 2015 United States Dollars

Parameter	Base value	Range	Source
Prevalence of HBsAg	0.062	0.057–0.067	[21] with 95% CI
Prevalence of HBeAg carrier if HBsAg+	0.307	0.273–0.341	30.7% of the HBsAg positive women [21]
Prevalence of HBV DNA > 200,000 IU per mL if HBsAg+	0.252	0.203–0.301	82% of the HBeAg positive women [22]
Probability of attending ANC at least 7 days before delivery can receive additional testing	0.946	0.944–0.998	SMRU data with 95% CI
Probability of attending ANC at least 3 m before delivery so can receive TDF	0.663	0.660–0.666	SMRU data with 95% CI
Proportion of women receiving TDF that would receive 5 m of treatment	0.720	0.716–0.724	SMRU data with 95% CI
Probability of clinic delivery after attending ANC	0.888	0.850–0.910	[21] SMRU data with 95% CI
Probability that infants birthed at home after attending ANC will receive HBV birth dose vaccine at the clinic (present within 12 h)	0.272	0.264–0.280	SMRU data with 95% CI
Probability of HBV perinatal infection for HBsAg+, HBeAg- mothers without vaccinations	0.110	0.070–0.150	[23–26] with low from [24] and high from [23]
Probability of HBV perinatal infection for HBeAg+ mothers without vaccinations	0.840	0.790–0.900	[23–25, 27] with low from [25] and high from [26]
Probability of HBV perinatal infection for HBsAg+, HBeAg- mothers with vaccinations	0.038	0.031–0.045	[28]
Probability of HBV perinatal infection for HBeAg+ mothers with vaccinations	0.210	0.143–0.278	[28–30]
Probability of HBV perinatal infection for HBsAg+, HBsAg- mothers with vaccinations and HBIG	0.010	0.001–0.030	[28]
Probability of HBV perinatal infection for HBeAg+ mothers with vaccinations and HBIG	0.180	0.100–0.260	[4]
Probability of HBV perinatal infection for HBsAg+, HBsAg- mothers with TDF, no HBIG	0.0025	0.0005–0.0055	Assumed based on midpoints of HBIG transmissions
Probability of HBV perinatal infection for HBeAg+ mothers with less than five months of TDF	0.0500	0.0070–0.0930	Assumed based on midpoints of HBIG transmissions
Probability of HBV perinatal infection for HBeAg+ mothers with five months of TDF	0.025	0.0001–0.050	Assumed based on lower points of HBIG transmissions
Probability of HBV perinatal infection for HBsAg+, HBeAg- mothers with TDF and vaccinations	0.0013	0.0003–0.0028	Assumed based on midpoints of TDF
Probability of HBV perinatal infection for HBeAg+ mothers with TDF and vaccinations	0.0128	0.0025–0.0280	Assumed based on midpoints of TDF
Probability of HBV perinatal infection for HBsAg+, HBeAg- mothers with TDF, vaccinations and HBIG	0.0003	0.00003–0.0006	Assumed based on midpoints of TDF and vaccinations
Probability of HBV perinatal infection for HBeAg+ mothers with TDF, vaccinations and HBIG	0.0005	0.0003–0.0013	Assumed based on midpoints of TDF and vaccinations
Probability of having a hepatic flare after stopping TDF	0.500	0.352–0.648	[31]
Probability that the hepatic flare requires treatment	0.308	0.163–0.453	[31]
Sensitivity of the RDT for HBsAg	0.980	0.631–1.000	Assumed based on [21]
Specificity of the RDT for HBsAg	0.989	0.969–1.000	Assumed based on [21]
Sensitivity of the confirmatory test for HBeAg	1.000	0.900–1.000	[32] with assumed range
Specificity of the confirmatory test for HBeAg	1.000	0.900–1.000	[32] with assumed range
Sensitivity of HBV DNA PCR	0.953	0.700–1.000	[33] with assumed range
Specificity of HBV DNA PCR	0.997	0.700–1.000	[33] with assumed range
Cost of HBV vaccinations	6.65	3.33–9.98	SMRU records + 50%. Cost of single vaccination at birth plus three doses of HBV diphtheria tetanus and pertussis combined vaccine given at 2, 4 and 6 months
Cost of RDT for HBsAg	1.3	0.65–1.96	SMRU records + 50%
Cost of confirmatory test	21.19	10.60–31.79	SMRU records + 50%
Cost of a PCR test	32.60	16.30	SMRU records + 50%
Cost per dose of HBIG	88.02	44.01–132.03	SMRU records + 50%
Cost per month of TDF	11.74	5.87–17.60	TDF Thailand bought
Cost for hepatic flare monitoring	6.85	3.42–10.27	ALT, Creatine and phosphate tests, twice each.
Cost of a hepatic flare	44.99	22.49–67.48	TDF for three additional months, five ALT tests

*ALT* Alanine aminotransferase, *ANC* antenatal care, *CI* Confidence interval, *DNA* Deoxyribonucleic acid, *EGA* estimated gestational age, *HBV* hepatitis B Virus, *HBIG* hepatitis B immunoglobulin, *HBeAg* hepatitis B envelope antigen, *HBsAg* hepatitis B surface antigen, *TDF* Tenofovir Disoproxil Fumarate, *PCR* Polymerase Chain Reaction, *SMRU* Shoklo Malaria Research Unit. Vaccinations, Birth dose and 3 follow up vaccinations

The effectiveness of maternal TDF to prevent perinatal transmission is dependent on the HBV DNA at the start of therapy and the number of months of TDF treatment before delivery. It was assumed that at least three months of TDF would be needed in order for it to have an effect on transmission. The probability of women attending ANC at least three months before delivery was 66.3% (95% CI 66.0–66.6%) Of the women that would receive TDF for at least three months, 72.0% would receive this for more than five months and therefore have a lower transmission probability. The probability of presenting at the clinic for the first time within 24 h after birth was calculated from those who attended ANC but delivered elsewhere (home or on the way to the clinic) but presented their infant at the SMRU clinic for birth weight measurement, 27.2% (95% CI 26.4–28.0%). These infants would still benefit from birth dose vaccination and could receive HBIG.

### Probabilities from the literature

The diagnostic accuracy of the RDT [34], confirmatory test [32], and PCR [33] were based on a literature review (Table 2). Initial screening for HBV was performed using a RDT with a reported sensitivity of 100% (63.1–100%) and specificity of 100% (98.9–100%) in the study population [35]. Molecular testing to establish the quantity of HBV DNA is unavailable in most RLS. A systematic review of HBeAg testing found it to be a possible proxy marker to detect the women of highest transmission risk with a pooled sensitivity of 92.0% (95% CI: 88.2–94.6%) [17]. The probability of attending ANC follow up, clinic delivery as well as follow up attendance for vaccination was extracted from previously published SMRU data [5, 21].

The probability of vertical transmission to the child was dependent on HBeAg status and interventions received (vaccination, HBIG, and/or TDF). We searched published literature to establish transmission rates for each strategy [4, 9, 23–26, 28–30, 36]. Our meta-analysis resulted in a perinatal transmission rate for HBsAg+/HBeAg- mothers of 11.0% (7.0–15.0%) and for HBsAg+/HBeAg+ mothers 84.0% (79.0–90.0%) [23–26] in the absence of any intervention. When all vaccinations were given (birth dose plus three follow up vaccinations), the probability of perinatal transmission was 3.8% (3.1–4.5%) for HBsAg+/HBeAg- mothers and 18% (10–26%) for HBsAg+/HBeAg+ mothers [4].

No studies have reported on perinatal transmission when providing (1) maternal TDF without HBV vaccination, (2) maternal TDF with HBV vaccination but without HBIG, or (3) maternal TDF with HBV vaccination and HBIG. Accordingly, these transmission probabilities were estimated. In these scenarios, the virus is unlikely to be transmitted if sufficient TDF is taken [37]. The probability of HBV transmission for HBsAg+ or HBeAg+ women that receive TDF without infant HBIG administration was estimated based on the midpoint of the transmission probability with the HBIG. Each subsequent probability was based on the midpoint of the transmission of the higher risk scenario. Of these unknown transmission rates, the highest risk was estimated for the maternal TDF without HBV vaccination. When maternal TDF with HBV vaccination is provided, the HBV transmission was estimated to drop considerably.

This risk of hepatic flare following the cessation of TDF treatment was taken from a study in Australia, which found that 50.0% (35.2–64.8%) of women would have a flare and 30.8% (16.3–45.3%) of those would require additional TDF treatment and lab tests [31]. This is in line with other published studies on post partum flare [38, 39].

### Cost parameters

Unit costs were taken from the 2019 financial records of the clinics and included those for diagnostic tests, vaccination and HBIG at the clinic. The price list from the external laboratory that the HBeAg test and HBV DNA test were sent to was used for those tests. All costs are reported in 2019 United States Dollars (USD). Unit costs were converted from Thai Baht into USD using the mid-year exchange rate for 2019 (1 Thai baht = 0.0326 USD) [40]. As all women were encouraged to deliver at the clinics, the cost of delivery was not included. All women given TDF were tested for phosphate, creatinine and ALT before birth and after delivery to determine whether a flare had occurred. The cost for treatment of a flare included 3 months of TDF and 5 ALT lab tests.

### Cost-effectiveness analysis

The method to analyze the cost effectiveness was adapted from a previously published study in the same setting [19]. For each of the six strategies, the total costs of the strategy as well as the expected perinatal HBV infections were calculated and plotted on a cost-effectiveness plane. After ordering the six strategies from the least to the most costly, any strategy that averted fewer perinatal infections than the previous less expensive strategy was considered dominated and thus removed. For the non-dominated strategies the incremental cost effectiveness ratio (ICER) was calculated using the following formula:

$$ \frac{C_B-{C}_A}{-\left({E}_B-{E}_A\right)} $$

where *C* is the cost and *E* is the infections averted for two strategies (*A* and *B*). Options with extended dominance were removed from the analysis. The results were compared with willingness to pay thresholds of one gross domestic product (GDP) per capita, US$1300, and one-half GDP per capita [41], US$650, for Myanmar.

To test the impact of each of the stated parameters on the ICER as well as whether a strategy was cost effective, a one way sensitivity analyses was conducted. Only the 15 parameters that had the greatest effect on the base case ICER were presented. To incorporate the uncertainty of parameter estimates over 10,000 sampling iterations from the distributions around the base case parameters, a probabilistic sensitivity analyses (PSA) was conducted with mean estimates and 95% credible intervals (CrIs). A cost-effectiveness acceptability curve was produced based on the PSA results that reports the likelihood of cost effectiveness of an intervention at different willingness-to-pay thresholds.

## Results

Table 3 presents the results for the base case analyses and CrIs from the PSA. The base results showed a range of costs from US$19,639–42,275 for the cohort with *Vaccine only* as the least expensive strategy resulting in the most infections and with *HBIG after RDT* as the most costly strategy with the least infections. This is equivalent to a cost per woman attending the clinic ranging from US$6.1 to US$13.2. The number of infections decreased from 22 in the *Vaccine only* scenario to 7 for the *TDF after RDT* scenario. With an ICERs of US$980, only the *TDF after HBeAg test* strategy was cost-effective for the one GDP per capita threshold; though this would not be considered cost-effective with the lower threshold of one-half GDP per capita. The *TDF after high HBV DNA* and *HBIG & TDF after high HBV DNA*, and *HBIG after RDT* strategies were all dominated because they cost more money than the *TDF after HBeAg test* strategy while averting the same or fewer infections. With an US$2489, the *TDF after RDT* scenario could be cost-effective if the willingness-to-pay threshold were higher.

**Table 3:** Base case results (95% Credible Intervals) for cohort of 3200 women. All costs are in 2019 United States Dollars (US$)

Strategy	Cost	Incremental cost	Infections	Infections averted	ICER
*Vaccine only*	$ 19,639 (11,202–30,258)	base	22 (17–27)	base	base
*TDF after HBeAg test*	$ 30,254 (21172–41,413)	$ 10,615 (7539–13,952)	11 (9–14)	11 (7–14)	$ 980 (689–1454)
*TDF after PCR*	$ 31,715 (22264–43,001)	dominated	11 (9–16)	dominated	dominated
*HBIG & TDF after PCR*	$ 34,791 (24915–46,417)	dominated	11 (9–15)	dominated	dominated
*TDF after RDT*	$ 39,845 (28439–52,958)	$ 9591 (4533–16,108)	7 (5–10)	4 (3–5)	$ 2489 (1267–4410)
*HBIG after RDT*	$ 42,275 (29,145–58,319)	dominated	17 (12–22)	dominated	dominated

*DNA* Deoxyribonucleic acid, *HBeAg* hepatitis B envelope antigen, *HBV* hepatitis B virus, *ICER* incremental cost-effectiveness ratio, *RDT* Rapid diagnostic test, *TDF* Tenofovir Disoproxil Fumarate

**Fig. 2:**
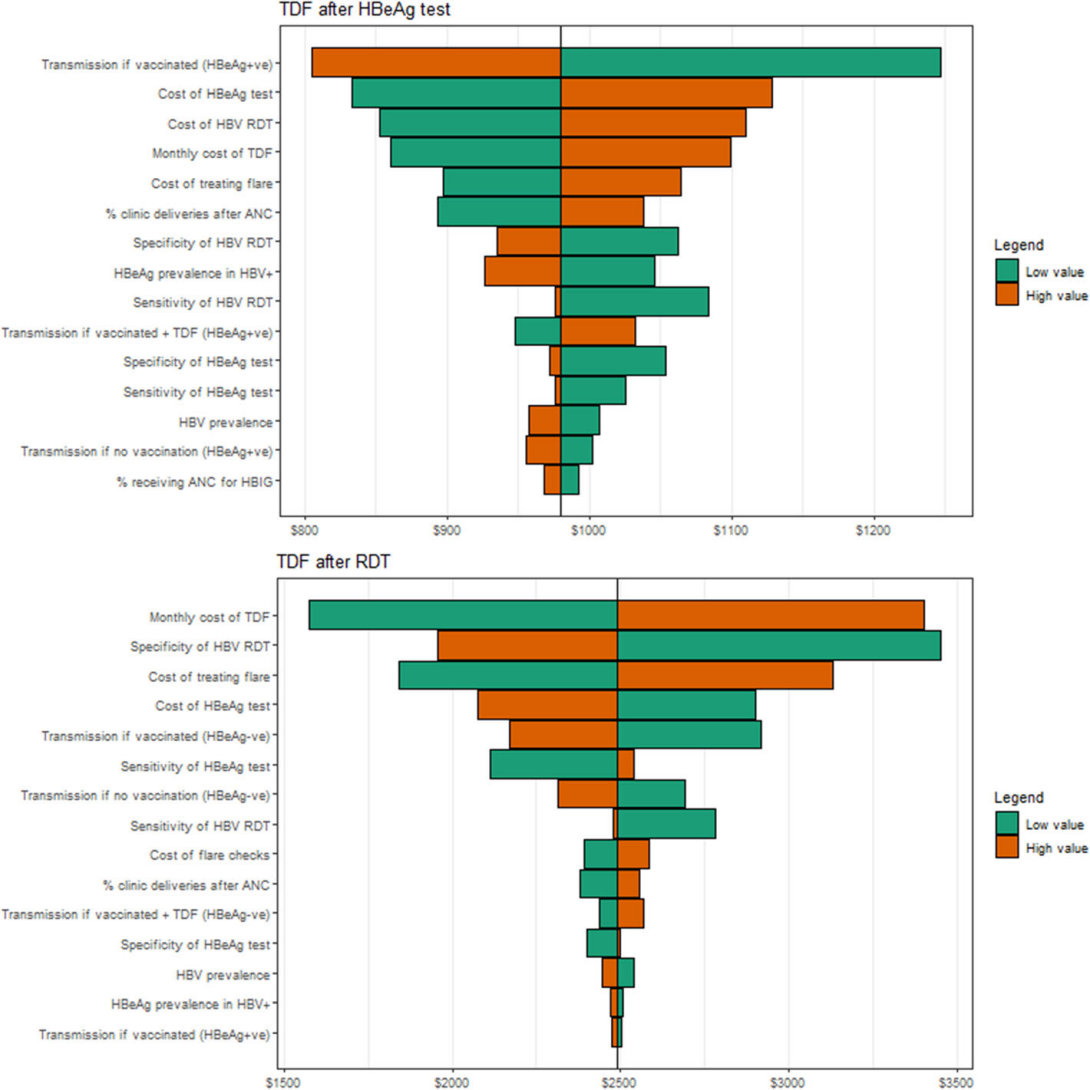
Results from the one-way sensitivity analysis. Impact of using the low and high values on the resulting incremental cost-effectiveness ratio. ANC, antenatal care; HBeAg, hepatitis B envelope antigen; HBV, hepatitis B virus; RDT, Rapid diagnostic test; TDF, Tenofovir Disoproxil Fumarate

The one-way sensitivity analysis (Fig. 2) demonstrated that the results were reasonably robust to changes in single parameter values with no values pushing the ICER for *TDF after HBeAg test* over US$1300 or under US$650. The monthly cost of TDF had a large impact on the cost-effectiveness of both *TDF after HBeAg test* and *TDF after RDT*. Other parameters with the largest impact on the ICER were also related to the probability of transmission if HBeAg positive and vaccinated, and the cost of a HBeAg test, HBV RDT, and treating a flare. Many of these parameters were also influential for the cost-effectiveness of *TDF after RDT* scenario, which was also influenced by the specificity of the HBV RDT.

**Fig. 3:**
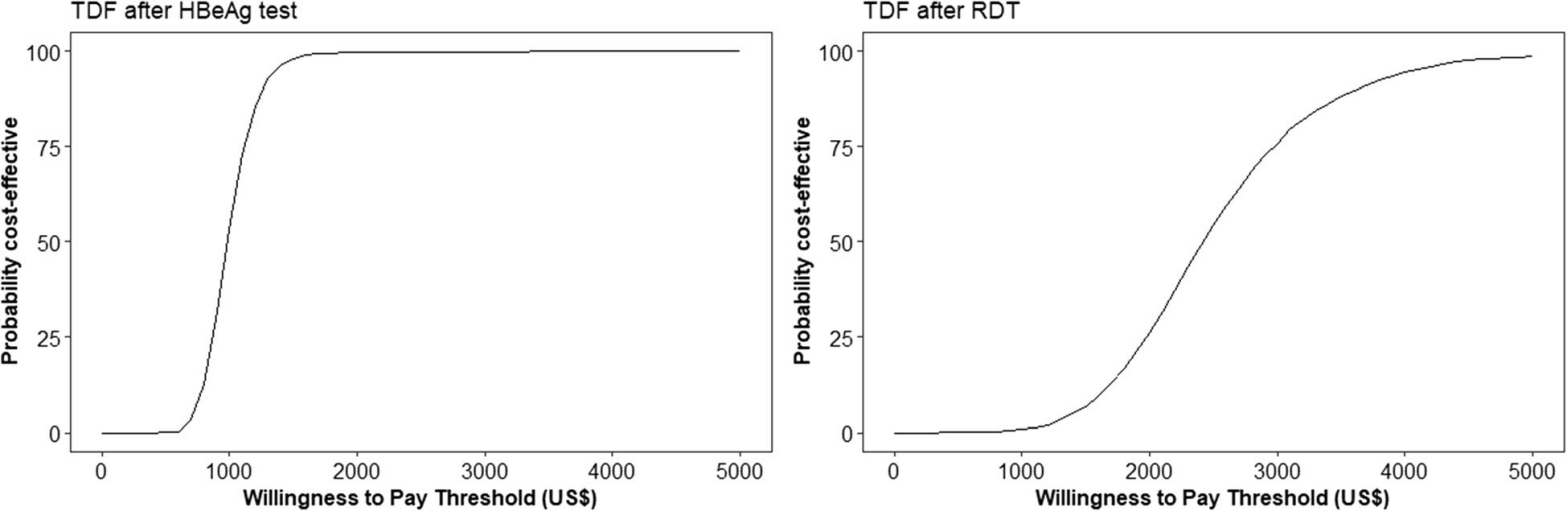
Cost-effectiveness acceptability curves in 2019 United States dollars (US$). The left panel is in comparison to the vaccine only strategy while the right panel is compared to the TDF after HBeAg test strategy. HBeAg, hepatitis B envelope antigen; TDF, Tenofovir Disoproxil Fumarate

The PSA showed that, at a willingness to pay threshold of US$1300 per infection averted, *TDF after HBeAg* had a 93% likelihood of being cost effective, Fig. 3. Decreasing this willingness to pay threshold to US$650 per infection averted, results in a drop of the likelihood of cost-effectiveness of this scenario to 1.3%.

## Discussion

Our results indicate that only three strategies for the prevention of mother to child transmission of hepatitis B have the potential to be cost-effective in a RLS, depending on the willingness to pay threshold. *Vaccine only* is the standard of care in most settings and the least costly option with the highest number of infections. With an ICER of US$980, the *TDF after HBeAg test* strategy was cost-effective using the willingness-to-pay threshold of 1 GDP per capita for Myanmar (US$1300) but not for the lower threshold of one-half GDP per capita (US$650). There is a significant gap in the willingness-to-pay threshold for Thailand (US$5058) a middle-income country, compared to its neighbour Myanmar, a low-income country. It is important to note that the threshold is typically tied to disability-adjusted life-years (DALYs) averted, which are not calculated in our model. Consequently, if each infection prevented averts more DALYs, then the ICER would decrease. In RLSs with a higher willingness to pay threshold, then the *TDF after RDT* strategy would be a cost-effective option with an ICER of US$2489. If the HBeAg status of the mother could be established with an RDT, which is inexpensive, on site and easy to perform in any setting, this could increase the chances of TDF implementation after HBeAg screening [8]. Alternatively, semiquantitative testing of serum HBeAg concentration may be a more realistic and cost-effective option in place of HBV DNA [42]. Moreover, neither scenario includes the HBIG, which would is less feasible in RLS because it requires a cold chain, has a high cost, and requires birth at a clinic [43]. In Myanmar, facility-based childbirth only reached 37% in 2015–16 [44], indicating the importance of prevention methods that apply to populations giving birth at home.

Other published economic evaluations of HBV preventive strategies during pregnancy have shown similar outcomes with a high cost effectiveness of TDF treatment after RDT, where it becomes more cost effective if there are ways to detect the women at highest risk of transmission [16, 18]. A previous study on the Thai-Myanmar border showed that HBIG usage was not cost effective in a RLS due to the high costs of the HBeAg test and HBIG [19]. Vaccination, birth dose in combination with three follow up vaccinations, is considered highly cost effective but remains challenging due to low completion in marginalized populations [8, 19]. Vaccination practices have improved in high resource settings but in RLS there is a documented completion of vaccination of approximately 50% in this area and in Chiang Mai Province for migrants [5]. However, HBV birth dose completion can be improved in RLS with coverage possible after a home birth if the infant is presented to a clinic within 12 h. Over the past years, the probability that infants birthed at home after attending ANC presented at the clinic within 12 h after delivery to receive HBV birth dose vaccine has increased from 15% in 2016 to 27% in the current analyses [19]. This increases the opportunities for HBV prevention services.

In the one-way sensitivity analysis, the monthly cost of TDF had a large impact on the cost-effectiveness of both *TDF after HBeAg test* and *TDF after RDT*. The total cost of TDF treatment is dependent on how early in the pregnancy women attend the clinic and the number of months of TDF treatment that is required to prevent transmission. The current guidelines recommend TDF treatment continue for one month post partum [45]. If the TDF treatment is initiated later in pregnancy, the months of treatment could shorten from eight to five months. This would be cheaper but might increase transmission probability.

The benefits across all scenarios would increase if disability-adjusted life years averted were included in a lifetime model. Approximately 85–90% of the people that acquire their HBV infection in infancy develop chronic HBV infection. Chronic infection has a 15–40% risk of developing complications, the most serious of which are liver cirrhosis, liver failure and hepatocellular carcinoma (HCC) [46, 47]. Approximately 1% of the chronically HBV infected will develop HCC which has a mortality of 40% [48]. Overall, HBV infection in infancy has a life-long mortality of approximately 25%; ie, for every 4 infections averted, 1 death is averted, which would translate into high numbers of disability-adjusted life years averted. Morbidity and mortality can be averted by antiviral treatment but in RLS this remains the main challenge as the costs of these medicines are not supported by governments or health insurance providers, leaving large out-of-pocket expenses for already marginalized population [49].

In most high resource settings, the HBV prevalence is low which limits the cost effectiveness of strategies that are highly cost effective in a setting with a high HBV prevalence. Earlier studies have indicated that maternal screening of all women in low prevalence setting is not cost effective compared to screening of high risk groups [50]. In high prevalence settings, the number needed to test is lower which makes universal maternal screening more cost effective [16, 18].

Our analysis has several limitations that need to be taken into account. The first group of limitations relates to uncertainties in our model parameters. Six of the transmission rates were estimated due to lack of data. Additionally, long term effects of HBV infection are not taken into account. In our model we assumed that women adhere to TDF treatment completely which might not be true. However, TDF has a long half life of 87 h in peripheral blood mononuclear cells, and 96 h in hepatocytes which makes it a pharmacologically “forgiving” medicine in the context of poor adherence [51]. Lastly in our model we assumed the infants would receive all HBV vaccinations, birth dose and 3 follow up vaccines, which is very challenging in RLS and especially in migrants. However, recent publications suggest that the uptake of the birth dose vaccination is high and documentation declines at the subsequent vaccinations [5]. As the birth dose vaccination is assumed to have the greatest impact on prevention of MTCT, the effect of a decreased uptake of follow up vaccinations is likely to have limited impact on our model. It is argued that TDF could be used for HBIG replacement (in order to avoid cold chain and reduce costs). What we don’t know is if TDF permits leniency in the window for vaccination of the birth dose. This would be helpful to establish since homebirth and delayed presentation of the child to health servicesare a RLS problem. TDF through pregnancy and childbirth might not only negate the need for HBIG but it might additionally allow children to be vaccinated at the first opportunity after birth outside of the current timeframe of 12–24 h. This analysis considered only current practice and costs based in Thailand. TDF is now off patent and cheaper brands are available in Myanmar making TDF strategies more realistic as these drugs cost approximately 50% less than TDF purchased from Thailand used in this analyses.

## Conclusion

This study represents a cost-effectiveness model of HBV PMTCT in a resource limited setting. The results demonstrate that while *vaccine only* is the least expensive strategy, it also leads to the most infections. *TDF after HBeAg test* and *TDF after RDT* strategies have the potential to be cost-effective with ICERs of US$980 and US$2489, respectively. Barriers to implementing these strategies remain costs of TDF treatment and reliable RDT. The most feasible strategy to prevent more HBV infections than *Vaccine only* in RLS seems to be *TDF after RDT* which would be easy to achieve in more rural settings.

## Supplementary Information

**Additional file 1: **Details of Strategy 1: *Vaccine only.*

**Additional file 2: **Details of Strategy 2: *TDF after HBeAg test.*

**Additional file 3: **Details of Strategy 3: *TDF after PCR.*

**Additional file 4: **Details of Strategy 4: *HBIG & TDF after PCR.*

**Additional file 5: **Details of Strategy 5: *TDF after RDT.*

**Additional file 6: **Details of Strategy 6: *HBIG after RDT.*

## Data Availability

The datasets used and/or analysed during the current study are available from the corresponding author on reasonable request.

## Abbreviations

ALT: Alanine transaminase
ANC: Antenatal care
BD: Birth dose
CrIs: Credible intervals
DNA: Deoxyribonucleic acid
GDP: Gross domestic product
HBeAg: Hepatitis B envelope antigen
HBIG: Hepatitis B immunoglobulins
HBsAg: Hepatitis B surface antigen
HBV: Hepatitis B Virus
HCC: Hepatocellular carcinoma
HIV: Human immunodeficiency virus
ICER: Incremental cost effectiveness ratio
MKT: Mawker Thai
MLA: Maela
MTCT: Mother to child transmission
PCR: Polymerase chain reaction
PSA: Probabilistic sensitivity analyses
RDT: Rapid diagnostic test
RLS: Resource limited setting
SMRU: Shoklo Malaria Research Unit
TDF: Tenofovir Disoproxil Fumarate
USD: United States Dollars
WPA: Wang-Pha

## References

[1] Pan CQ, Duan ZP, Bhamidimarri KR, Zou HB, Liang XF, Li J, Tong MJ (2012). An algorithm for risk assessment and intervention of mother to child transmission of hepatitis B virus. Clin Gastroenterol Hepatol.

[2] Li Z, Hou X, Cao G (2015). Is mother-to-infant transmission the most important factor for persistent HBV infection?. Emerg Microbes Infect.

[3] Childs L, Roesel S, Tohme RA (2018). Status and progress of hepatitis B control through vaccination in the South-East Asia region, 1992-2015. Vaccine.

[4] Pan CQ, Duan Z, Dai E, Zhang S, Han G, Wang Y, Zhang H, Zou H, Zhu B, Zhao W (2016). Tenofovir to prevent hepatitis B transmission in mothers with high viral load. N Engl J Med.

[5] Bierhoff M, Pinyopornpanish K, Pinyopornpanish K, Tongprasert F, Keereevijit A, Rijken M, Chu CS, Nosten F, Perfetto J, van Vugt M (2019). Retrospective Review of Documentation Practices of Hepatitis B Immunoglobulin, Birth Dose, and Vaccination at the Hospital of Birth, in Thai Nationals and Migrants in Northern Thailand. Open Forum Infect Dis.

[6] Li Y, Wang J, Yu Y, Qiu C, Li Z, Ling Q, Zhang G, Li L, Gong Y, Lu Q, Cao L, Gu T, Wang X, Zhang M, Zhang Q, Zhang H, Xu B, Shao L, Pu Y, Zhang W. Maternal antiviral treatment safeguards infants from hepatitis B transmission in contingencies of delayed immunoprophylaxis. Liver Int. 2020;40(10):2377–84. 10.1111/liv.14479. 10.1111/liv.14479 32304160

[7] Li XM, Yang YB, Hou HY, Shi ZJ, Shen HM, Teng BQ, Li AM, Shi MF, Zou L (2003). Interruption of HBV intrauterine transmission: a clinical study. World J Gastroenterol.

[8] Funk AL, Lu Y, Yoshida K, Zhao T, Boucheron P, van Holten J, Chou R, Bulterys M, Shimakawa Y (2021). Efficacy and safety of antiviral prophylaxis during pregnancy to prevent mother-to-child transmission of hepatitis B virus: a systematic review and meta-analysis. Lancet Infect Dis.

[9] Jourdain G, Ngo-Giang-Huong N, Harrison L, Decker L, Khamduang W, Tierney C, Salvadori N, Cressey TR, Sirirungsi W, Achalapong J (2018). Tenofovir versus placebo to prevent perinatal transmission of hepatitis B. N Engl J Med.

[10] Lee YS, Lee HS, Kim JH, et al. Role of tenofovir disoproxil fumarate in prevention of perinatal transmission of hepatitis B virus from mother to child: a systematic review and meta-analysis. Korean J Intern Med. 2021;36(1):76–85. 10.3904/kjim.2018.465. 10.3904/kjim.2018.465 PMC7820648 31878771

[11] Gao F, Zhang WT, Lin YY, Wang WM, Xu N, Bai GQ (2019). Early start of Tenofovir treatment achieves better viral suppression in pregnant women with a high HBV viral load: a real-world prospective study. Infect Drug Resist.

[12] Bierhoff M, Nelson KE, Guo N, et al. Prevention of mother-to-child transmission of hepatitis B virus: protocol for a one-arm, open-label intervention study to estimate the optimal timing of tenofovir in pregnancy. BMJ Open. 2020;10(9):e038123. 10.1136/bmjopen-2020-038123. 10.1136/bmjopen-2020-038123 PMC7488796 32928858

[13] Dionne-Odom J, Njei B, Tita ATN (2018). Elimination of vertical transmission of hepatitis B in Africa: a review of available tools and new opportunities. Clin Ther.

[14] Fan L, Owusu-Edusei K, Schillie SF, Murphy TV (2016). Cost-effectiveness of active-passive prophylaxis and antiviral prophylaxis during pregnancy to prevent perinatal hepatitis B virus infection. Hepatology (Baltimore, Md).

[15] Wang W, Wang J, Dang S, Zhuang G (2016). Cost-effectiveness of antiviral therapy during late pregnancy to prevent perinatal transmission of hepatitis B virus. PeerJ.

[16] Cui F, Woodring J, Chan P, Xu F (2018). Considerations of antiviral treatment to interrupt mother-to-child transmission of hepatitis B virus in China. Int J Epidemiol.

[17] Pauline Boucheron KY, Ying Lu, Tianshuo Zhao, Anna-Louise Funk, Judith van Holten, Yvan Hutin, Marc Bulterys, Yusuke Shimakawa: Performance of hepatitis B e antigen (HBeAg) test, as an alternative to HBV DNA, to assess eligibility for initiating antiviral therapy during pregnancy to prevent mother-to-child transmission of hepatitis B: a systematic review and meta-analysis. In: Boston: CROI. webcast; 2020.

[18] Mokaya J, Burn EAO, Tamandjou CR, Goedhals D, Barnes EJ, Andersson M, Pinedo-Villanueva R, Matthews PC (2019). Modelling cost-effectiveness of tenofovir for prevention of mother to child transmission of hepatitis B virus (HBV) infection in South Africa. BMC Public Health.

[19] Devine A, Harvey R, Min AM, Gilder MET, Paw MK, Kang J, Watts I, Hanboonkunupakarn B, Nosten F, McGready R (2017). Strategies for the prevention of perinatal hepatitis B transmission in a marginalized population on the Thailand-Myanmar border: a cost-effectiveness analysis. BMC Infect Dis.

[20] R Core Team (2020). R: a language and environment for statistical computing.

[21] Bierhoff M, Angkurawaranon C, Myat Min A, Gilder ME, Win Tun N, Keereevijitt A, Kyi Win A, Win E, Carrara VI, Brummaier T et al: Maternal hepatitis B infection burden, Comorbidity and Pregnancy Outcome in a Low-Income Population on the Myanmar-Thailand Border: A Retrospective Cohort Study**.** J Pregnancy 2019, 2019:8435019. 10.1155/2019/8435019 PMC6413357 30931155

[22] Pan CQ, Mi LJ, Bunchorntavakul C, Karsdon J, Huang WM, Singhvi G, Ghany MG, Reddy KR (2012). Tenofovir disoproxil fumarate for prevention of vertical transmission of hepatitis B virus infection by highly viremic pregnant women: a case series. Dig Dis Sci.

[23] Beasley RP, Trepo C, Stevens CE, Szmuness W (1977). The e antigen and vertical transmission of hepatitis B surface antigen. Am J Epidemiol.

[24] Nayak NC, Panda SK, Zuckerman AJ, Bhan MK, Guha DK (1987). Dynamics and impact of perinatal transmission of hepatitis B virus in North India. J Med Virol.

[25] Xu ZY, Liu CB, Francis DP, Purcell RH, Gun ZL, Duan SC, Chen RJ, Margolis HS, Huang CH, Maynard JE (1985). Prevention of perinatal acquisition of hepatitis B virus carriage using vaccine: preliminary report of a randomized, double-blind placebo-controlled and comparative trial. Pediatrics.

[26] Okada K, Kamiyama I, Inomata M, Imai M, Miyakawa Y (1976). E antigen and anti-e in the serum of asymptomatic carrier mothers as indicators of positive and negative transmission of hepatitis B virus to their infants. N Engl J Med.

[27] Beasley RP, Hwang LY, Lee GC, Lan CC, Roan CH, Huang FY, Chen CL (1983). Prevention of perinatally transmitted hepatitis B virus infections with hepatitis B immune globulin and hepatitis B vaccine. Lancet (London, Engl).

[28] Wang J, Liu J, Qi C, Yan T, Cao F, Jin L, He Y, Yang Y, Zhang S, Chen T (2015). Efficacy of tenofovir disoproxil fumarate to prevent vertical transmission in mothers with lamivudine-resistant HBV. Antivir Ther.

[29] Anderson EJ, Daugherty MA, Pickering LK, Orenstein WA, Yogev R (2018). Protecting the community through child vaccination. Clin Infect Dis.

[30] Ekra D, Herbinger KH, Konate S, Leblond A, Fretz C, Cilote V, Douai C, Da Silva A, Gessner BD, Chauvin P (2008). A non-randomized vaccine effectiveness trial of accelerated infant hepatitis B immunization schedules with a first dose at birth or age 6 weeks in cote d'Ivoire. Vaccine.

[31] Nguyen V, Tan PK, Greenup AJ, Glass A, Davison S, Samarasinghe D, Holdaway S, Strasser SI, Chatterjee U, Jackson K (2014). Anti-viral therapy for prevention of perinatal HBV transmission: extending therapy beyond birth does not protect against post-partum flare. Aliment Pharmacol Ther.

[32] Ratnam S, Tobin AM (1987). Comparative evaluation of commercial enzyme immunoassay kits for detection of hepatitis B seromarkers. J Clin Microbiol.

[33] Dai CY, Yu ML, Chen SC, Lin ZY, Hsieh MY, Wang LY, Tsai JF, Chuang WL, Chang WY (2004). Clinical evaluation of the COBAS Amplicor HBV monitor test for measuring serum HBV DNA and comparison with the Quantiplex branched DNA signal amplification assay in Taiwan. J Clin Pathol.

[34] Banks T, Kang J, Watts I, Tyrosvoutis ME, Min AM, Tun NW, Keereecharoen L, Simmawong W, Wanyatip S, Hanboonkunupakarn B (2016). High hepatitis B seroprevalence and risk factors for infection in pregnant women on the Thailand-Myanmar border. J Infect Dev Ctries.

[35] World Health Organization, GUIDELINES ON HEPATITIS B AND C TESTING, February 2017.

[36] Wong VC, Ip HM, Reesink HW, Lelie PN, Reerink-Brongers EE, Yeung CY, Ma HK (1984). Prevention of the HBsAg carrier state in newborn infants of mothers who are chronic carriers of HBsAg and HBeAg by administration of hepatitis-B vaccine and hepatitis-B immunoglobulin. Double-blind randomised placebo-controlled study. Lancet (London, England).

[37] Jourdain G, Ngo-Giang-Huong N, Cressey TR, Hua L, Harrison L, Tierney C, Salvadori N, Decker L, Traisathit P, Sirirungsi W (2016). Prevention of mother-to-child transmission of hepatitis B virus: a phase III, placebo-controlled, double-blind, randomized clinical trial to assess the efficacy and safety of a short course of tenofovir disoproxil fumarate in women with hepatitis B virus e-antigen. BMC Infect Dis.

[38] ter Borg MJ, Leemans WF, de Man RA, Janssen HL (2008). Exacerbation of chronic hepatitis B infection after delivery. J Viral Hepat.

[39] Tan HH, Lui HF, Chow WC (2008). Chronic hepatitis B virus (HBV) infection in pregnancy. Hepatol Int.

[40] Current and Historical Rate Tables. XE Currency Table: THB - Thai Baht. https://www.xe.com/currencytables/?from=THB&date=2019-07-01. Accessed 17 June 2020.

[41] GDP per capita (current US$) for Myanmar. http://data.worldbank.org/indicator/NY.GDP.PCAP.CD?locations=MM. Accessed 17 June 2020.

[42] Chen P, Xie Q, Lu X, Yu C, Xu K, Ruan B, Cao H, Gao H, Li L (2017). Serum HBeAg and HBV DNA levels are not always proportional and only high levels of HBeAg most likely correlate with high levels of HBV DNA: a community-based study. Medicine.

[43] Cavallin F, Trevisanuto D, Thein A, Booth A, Arnolda G, Kumara D, Phyu U, Myint S, Moccia L (2020). Birthplace is a risk factor for exchange transfusion in outborn infants admitted for jaundice in Myanmar: a case-control study. J Matern-Fetal Neonatal Med.

[44] Ministry of Health and Sports (2017). Myanmar Demographic and Health Survey 2015–16.

[45] Thailand Practice Guideline for Management of Chronic Hepatitis B and C 2015. http://www.thasl.org/files/25.Thailand%20guideline%20for%20management%20of%20CHB%20%20and%20CHC%202015.pdf. Accessed 17 June 2020.

[46] Hamburg-Shields E, Prasad M (2020). Infectious hepatitis in pregnancy. Clin Obstet Gynecol.

[47] Fattovich G, Bortolotti F, Donato F (2008). Natural history of chronic hepatitis B: special emphasis on disease progression and prognostic factors. J Hepatol.

[48] Aggarwal R, Ghoshal UC, Naik SR (2003). Assessment of cost-effectiveness of universal hepatitis B immunization in a low-income country with intermediate endemicity using a Markov model. J Hepatol.

[49] Wait S, Kell E, Hamid S, Muljono DH, Sollano J, Mohamed R, Shah S, Mamun Al M, Abbas Z, Johnston J (2016). Hepatitis B and hepatitis C in southeast and southern Asia: challenges for governments. Lancet Gastroenterol Hepatol.

[50] Hahne SJ, Veldhuijzen IK, Wiessing L, Lim TA, Salminen M, Laar M (2013). Infection with hepatitis B and C virus in Europe: a systematic review of prevalence and cost-effectiveness of screening. BMC Infect Dis.

[51] Delaney WE, Ray AS, Yang H, Qi X, Xiong S, Zhu Y, Miller MD (2006). Intracellular metabolism and in vitro activity of tenofovir against hepatitis B virus. Antimicrob Agents Chemother.

