# Supplementary figures and images for: Tenofovir disoproxil fumarate in pregnancy for prevention of mother to child transmission of hepatitis B in a rural setting on the Thailand-Myanmar border: a cost-effectiveness analysis
Source: BMC Pregnancy Childbirth. 2021 Feb 22;21:157. doi: 10.1186/s12884-021-03612-z (PMC7901182; doi:10.1186/s12884-021-03612-z)

## Strategy 1

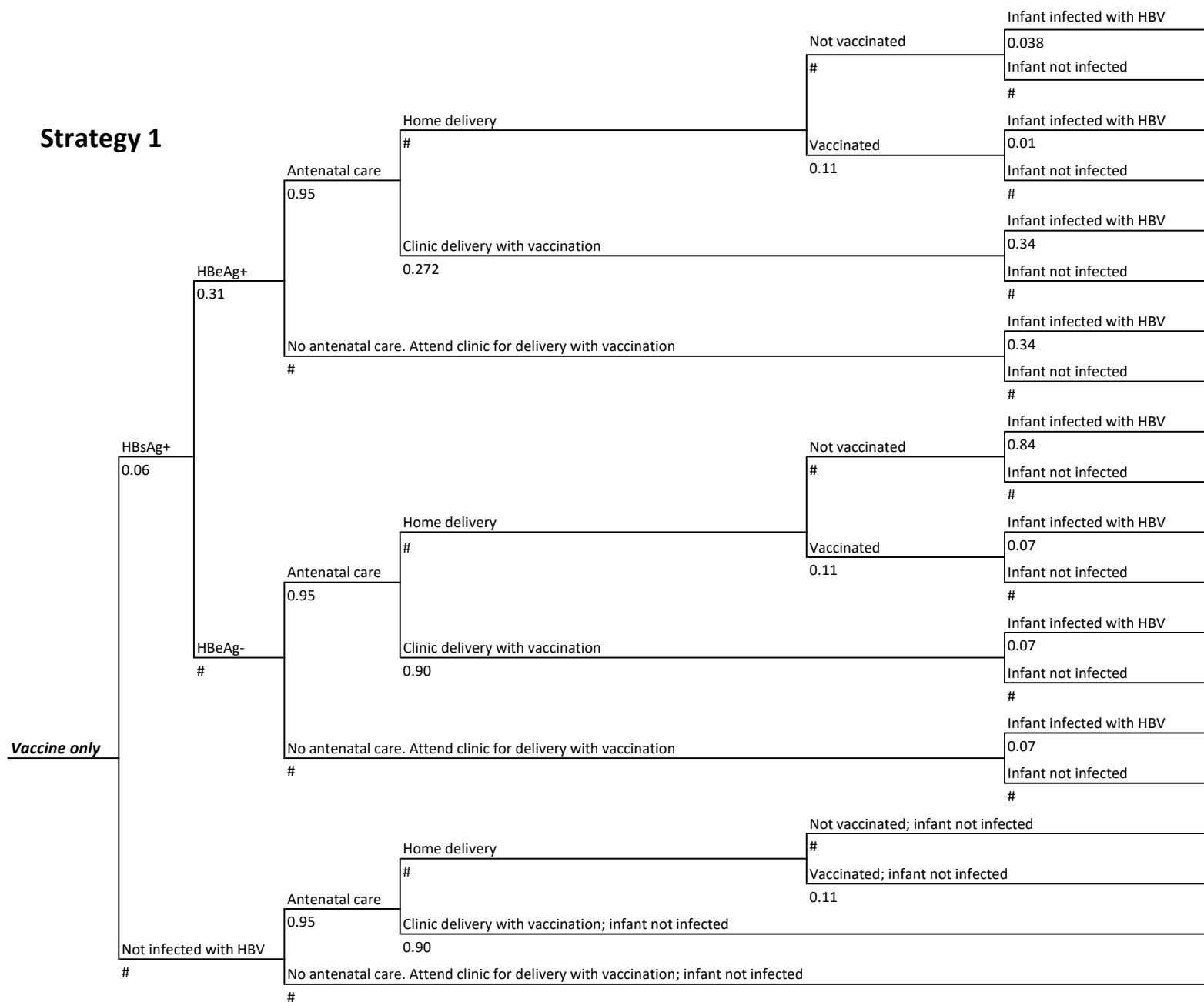

Supplement: Supplementary file 1 — Additional file 1: Details of Strategy 1: Vaccine only. [file 12884_2021_3612_MOESM1_ESM.pdf]

## Strategy 2

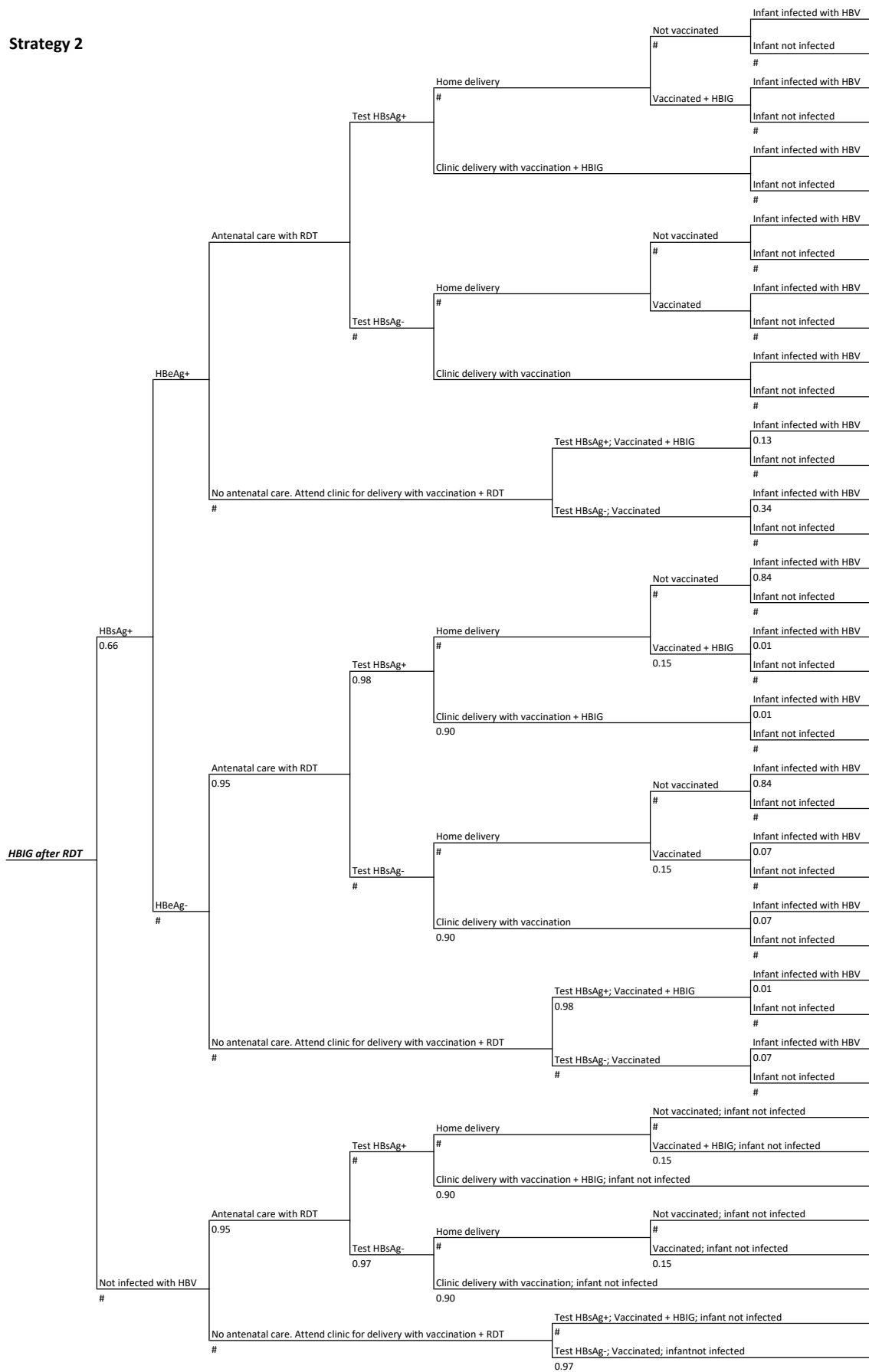

Supplement: Supplementary file 2 — Additional file 2: Details of Strategy 2: TDF after HBeAg test. [file 12884_2021_3612_MOESM2_ESM.pdf]

### Strategy 3

15% of these women have flare costs (probflare \* treatflare)

**TDF after RDT**

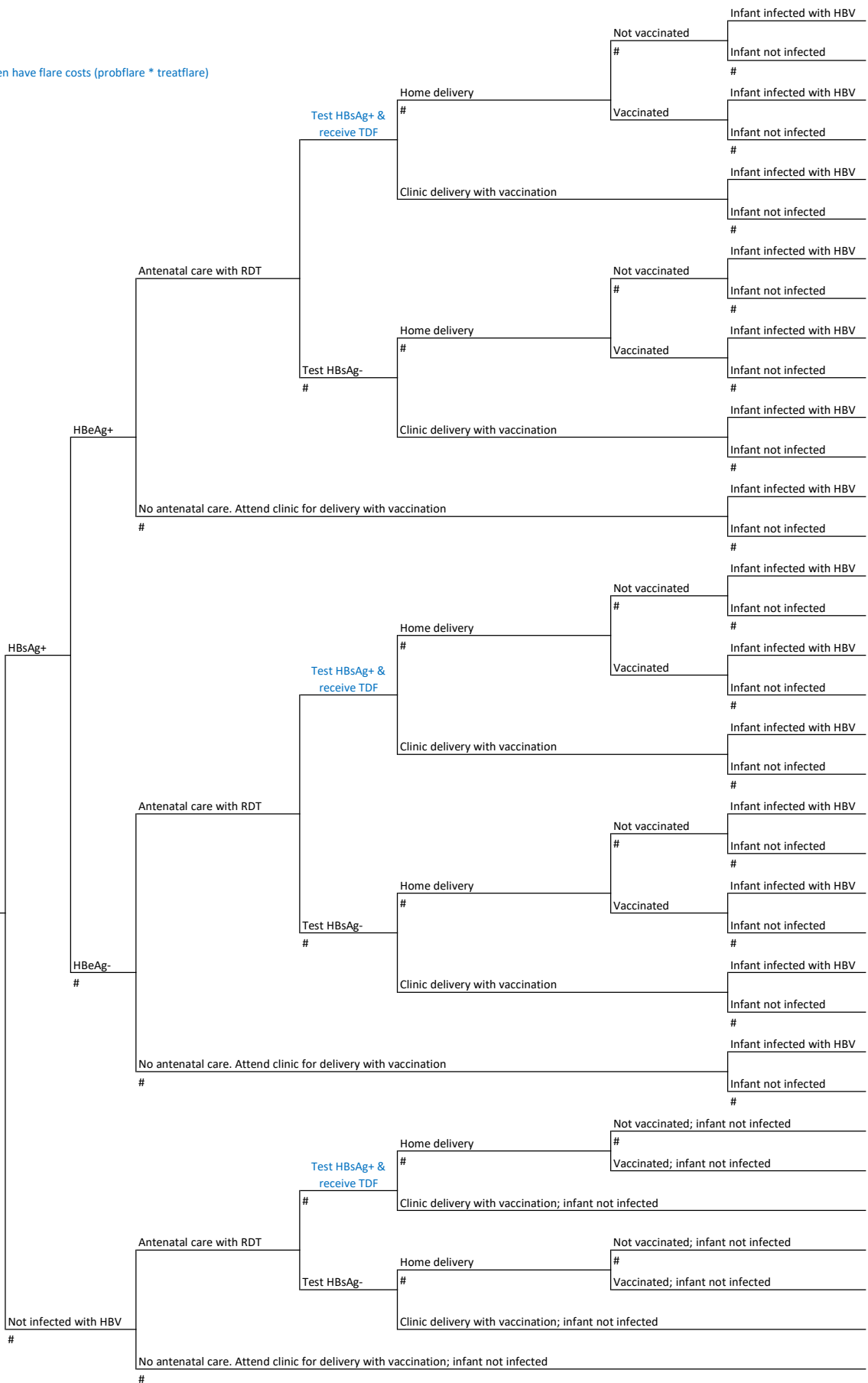

Supplement: Supplementary file 3 — Additional file 3: Details of Strategy 3: TDF after PCR. [file 12884_2021_3612_MOESM3_ESM.pdf]

## Strategy 4

15% of these women have flare costs (probflare \* treatflare)

TDF after  
confirmatory test

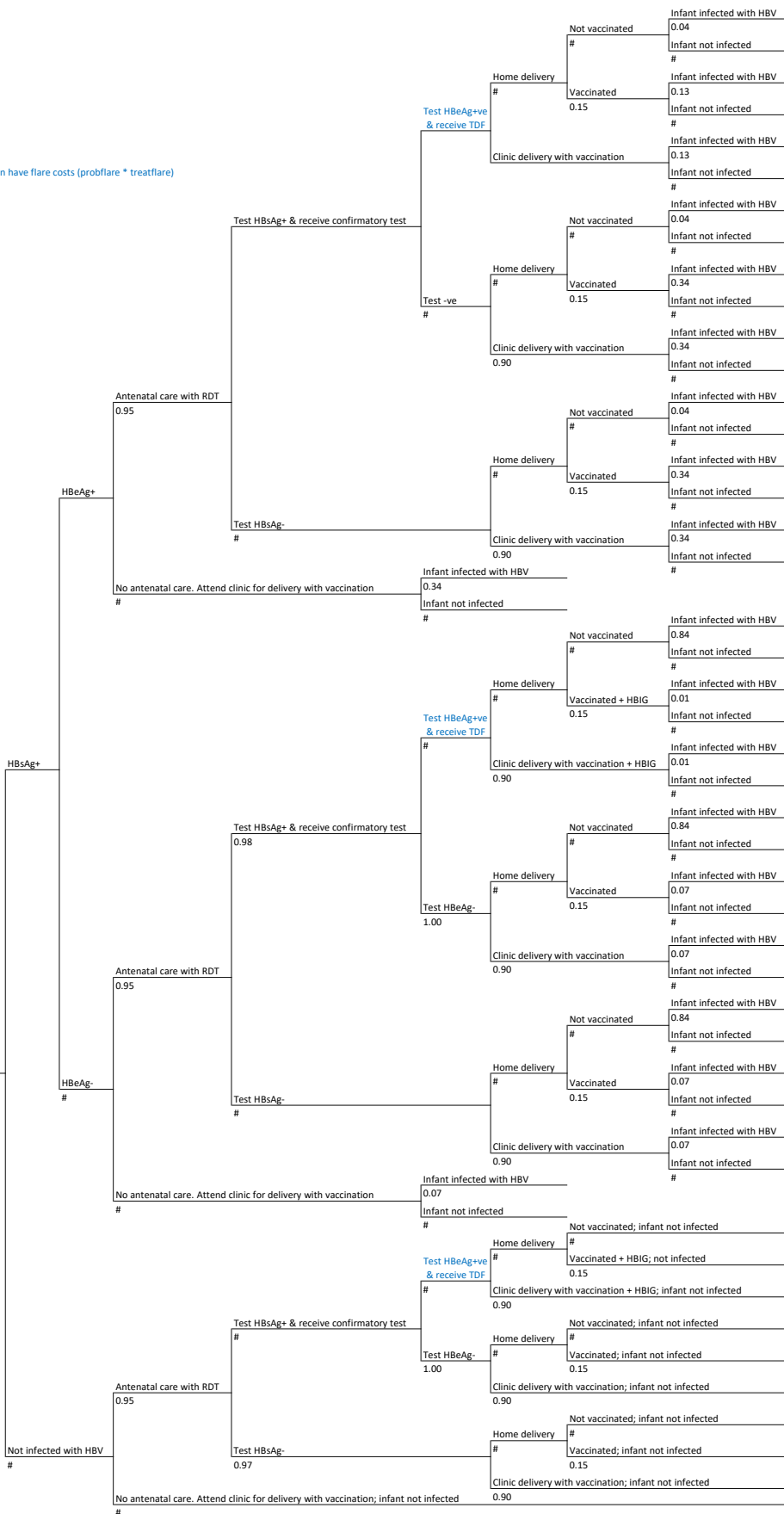

Supplement: Supplementary file 4 — Additional file 4: Details of Strategy 4: HBIG & TDF after PCR. [file 12884_2021_3612_MOESM4_ESM.pdf]

## Strategy 5

15% of these women have flare costs (proflare \* treatflare)

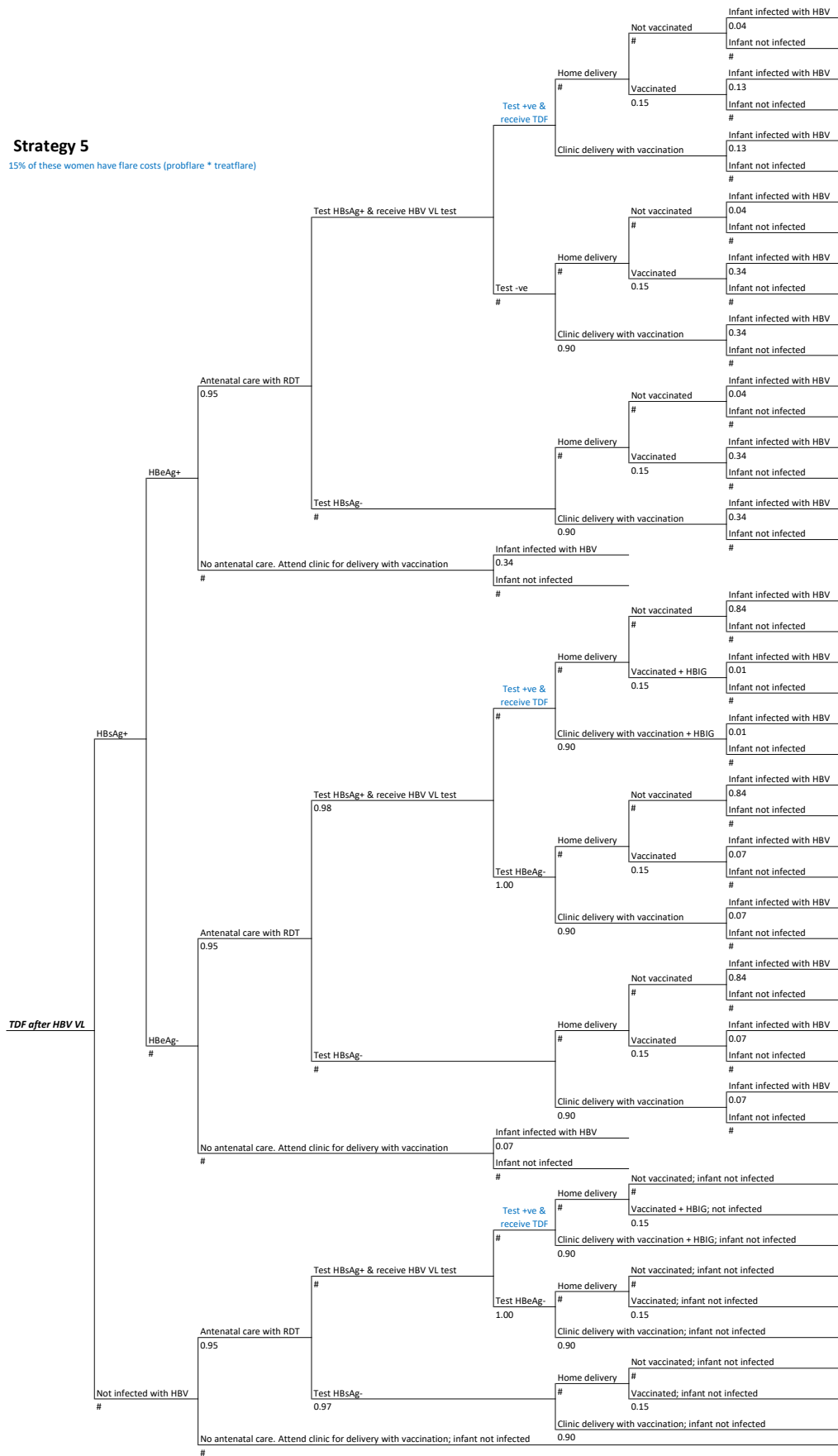

Supplement: Supplementary file 5 — Additional file 5: Details of Strategy 5: TDF after RDT. [file 12884_2021_3612_MOESM5_ESM.pdf]
