# Supplementary material for: Tenofovir disoproxil fumarate in pregnancy for prevention of mother to child transmission of hepatitis B in a rural setting on the Thailand-Myanmar border: a cost-effectiveness analysis
Source: BMC Pregnancy Childbirth. 2021 Feb 22;21:157. doi: 10.1186/s12884-021-03612-z (PMC7901182; doi:10.1186/s12884-021-03612-z)

Strategy 6

15% of these women have flare costs (probflare * treatflare)

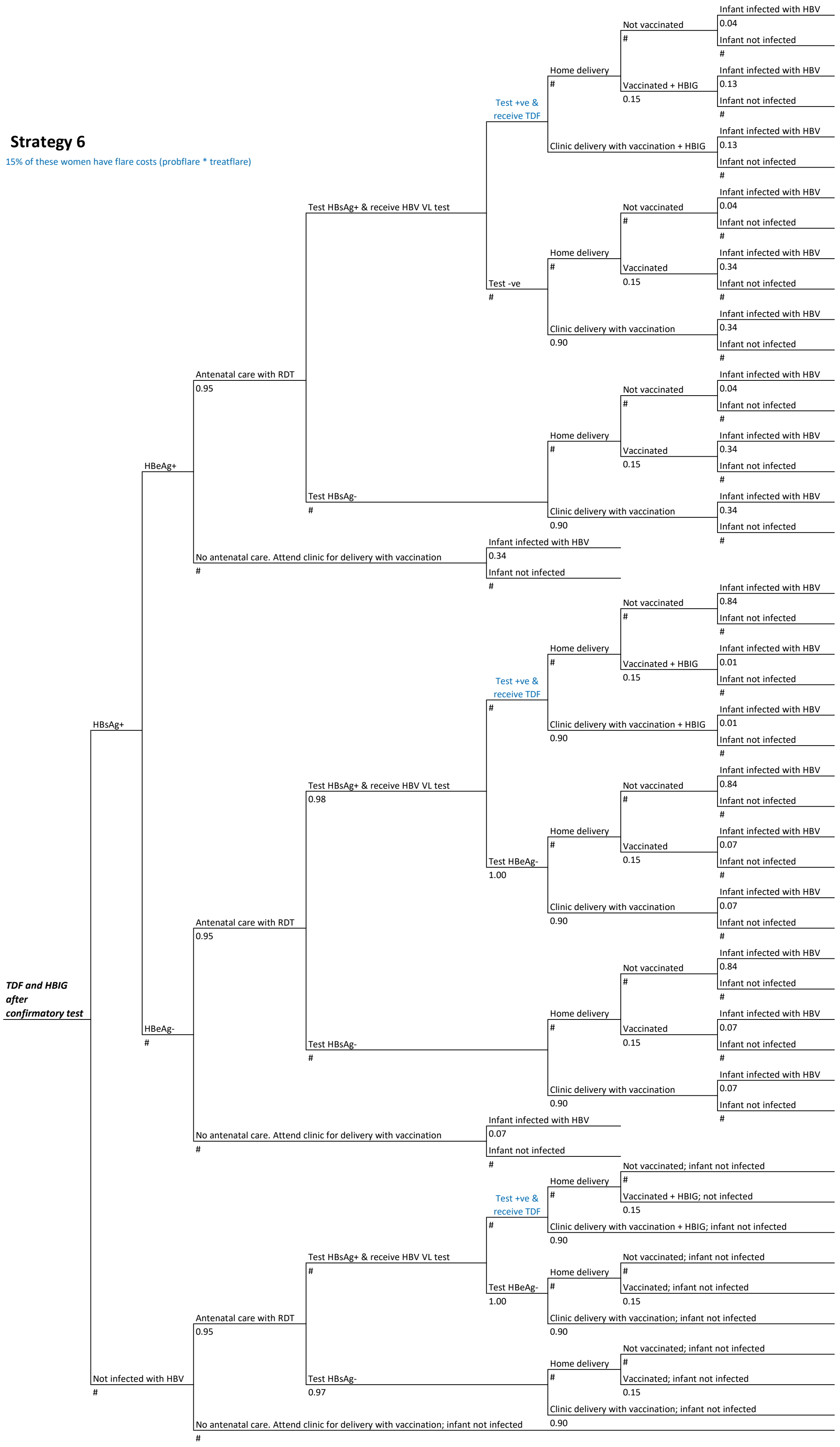

Supplement: Supplementary file 6 — Additional file 6: Details of Strategy 6: HBIG after RDT. [file 12884_2021_3612_MOESM6_ESM.pdf]
